# Effects of a Planned Web-Based Educational Intervention Based on the Health Belief Model for Patients With Ischemic Stroke in Promoting Secondary Prevention During the COVID-19 Lockdown in China: Quasi-Experimental Study

**DOI:** 10.2196/44463

**Published:** 2024-04-24

**Authors:** Zhuo Liu, Xin Sun, Zhen-Ni Guo, Ye Sun, Yi Yang, Xiuli Yan

**Affiliations:** 1 Stroke Center, Department of Neurology, The First Hospital of Jilin University, Changchun, China; 2 Neuroscience Research Center, Department of Neurology, The First Hospital of Jilin University, Changchun, China

**Keywords:** health belief model, health education, secondary prevention, stroke, medication adherence, patient education, web-based education, digital intervention, promotion, stroke patients, ischemic, prevention, quasi-experimental study, education, control group, health management, management, systolic blood pressure, blood pressure, effectiveness, medication adherence

## Abstract

**Background:**

Some common modified vascular risk factors remain poorly controlled among stroke survivors, and educational programs may help improve these conditions.

**Objective:**

This study aimed to evaluate the effect of a planned web-based educational intervention based on the health belief model (HBM) in promoting secondary prevention among patients with ischemic stroke.

**Methods:**

An evaluation-blinded quasi-experimental trial with a historical control group was conducted. Patients admitted from March to June 2020 were assigned to the historical control group, and patients admitted from July to October 2020 were assigned to the intervention group. The control group received routine health management. The intervention group received 6 additional sessions based on the HBM via Tencent Meeting, an audio and video conferencing application, within 3 months after discharge. Sessions were held every 2 weeks, with each session lasting approximately 40 minutes. These sessions were conducted in small groups, with about 8 to 10 people in each group. The primary outcomes were changes in blood pressure (BP), low-density lipoprotein cholesterol (LDL-C), hemoglobin A_1c_ (HbA_1c_), and the proportion of patients achieving the treatment target. The secondary outcomes were medication adherence, assessed with the Morisky Medicine Adherence Scale (MMAS), and disability, assessed with the modified Rankin scale.

**Results:**

In total, 315 patients experiencing their first-ever stroke were analyzed. More patients in the intervention group had controlled BP (41.9% vs 28.4%; adjusted odds ratio [aOR] 1.93; *P*=.01), LDL-C (83.1% vs 67.7%; aOR 2.66; *P*=.001), and HbA_1c_ (91.9% vs 83.9%; aOR: 3.37; *P*=.04) levels as well as a significant postintervention decrease in the systolic BP (adjusted *β* −3.94; *P*=.02), LDL-C (adjusted *β* −0.21; *P*=.008), and HbA_1c_ (adjusted *β* −0.27; *P*<.001), compared with control groups. Significant between-group differences were observed in medication adherence (79.4% vs 63.2%; aOR 2.31; *P*=.002) but not in favorable functional outcomes.

**Conclusions:**

A web-based education program based on the HBM may be more effective than current methods used to educate patients having strokes on optimal vascular risk factors and medication adherence.

## Introduction

According to the Global Burden of Disease Study 2019, stroke is the second leading cause of death worldwide [1]. It also presents a substantial challenge to the Chinese population, as it is the leading cause of mortality, long-term disability, and severe disease burden in China. Ischemic stroke accounts for the largest proportion of these cases [2,3]. Meanwhile, the mortality of ischemic stroke is lower than that of hemorrhagic stroke in middle-income countries; therefore, more attention needs to be directed toward patients who experienced ischemic stroke [4]. Data based on the China National Stroke Registries revealed that the stroke recurrence rate was approximately 12.5% within 12 months, which may lead to the deterioration of functional outcomes, decreased quality of life, and even increased mortality [5]. Therefore, it is necessary to achieve secondary prevention of ischemic stroke through lifestyle changes and medication intervention. A qualitative study found that stroke survivors perceived that using preventive medications was more important than modifying lifestyle behaviors [6]. It was previously reported that rational use of these drugs may reduce the risk of recurrent stroke and improve survival [7,8]. However, a systematic review of 22 studies showed that the pooled prevalence of medication nonadherence was 29% [9], and the risk factors remain poorly controlled among stroke survivors [10].

The health belief model (HBM) is widely used to guide health education, aiming to help participants develop, adopt, and practice healthy behaviors [11,12]. It emphasizes the subjective psychological process of individuals, suggesting that individuals with beliefs related to disease and health are more willing to adopt healthy behaviors. This theoretical model guides the health education for secondary prevention of stroke from six aspects: (1) helping patients identify their own risk factors for strokes (perceived susceptibility), (2) understanding the possible adverse consequences of stroke recurrence (perceived severity), (3) becoming aware of barriers (perceived barriers) and (4) benefits of secondary prevention(perceived benefits), (5) receiving information and encouragement through social media (cues to action), and (6) establishing confidence in their ability to engage in disease prevention (self-efficacy). When individuals are provided with these factors, they are more likely to adopt the recommended behaviors for secondary prevention.

In particular, the COVID-19 pandemic has had a negative impact on post–acute stroke care [13]. Care pathways focused on secondary prevention of cardiovascular disease may be disrupted, stroke follow-up may be delayed or reduced, and survivors may have difficulty accessing rehabilitation services [14,15]. China has implemented strict national policies to reduce the risk of exposure to the virus [16]. Staff shortages caused by a large number of medical staff supporting high-risk areas, quarantine efforts, and pathogen testing have led to a reduction in outpatient clinics and medical services available to patients having strokes [17]. Therefore, theory-based and implementable secondary prevention measures become particularly important to improve medication adherence and control of vascular risk factors, such as blood pressure (BP), hemoglobin A_1c_ (HbA_1c_), and low-density lipoprotein cholesterol (LDL-C) levels, within a reasonable range, for stroke survivors during lockdown.

This study aimed to investigate the effects of planned health education based on the HBM, compared to general health management, on improving medication adherence and controlling vascular risk factors in patients ischemic stroke 3 months after admission during the COVID-19 lockdown.

## Methods

### Study Design

This study was an assessor-blinded quasi-experimental trial with a historical control group. Consecutive participants admitted to the stroke center of the First Hospital of Jilin University were recruited. The hospital is located in the central part of Jilin Province, northeast China, with a population of 27 million and the highest age-standardized prevalence and incidence rate of stroke [5]. It also has the first province-wide stroke emergency map in China, named the Jilin Province Stroke Emergency Maps [18]. The transportation of patients between cities ensured the diversity of participants in our center. Patients admitted from March to June 2020 were assigned to the historical control group, and patients admitted from July to October 2020 were assigned to the intervention group.

### Ethical Considerations

This study was conducted in compliance with the principles of the Declaration of Helsinki. The study protocol was reviewed and approved by the ethical committee of the First Hospital of Jilin University (20K055-001) and was registered in the Chinese Clinical Trial Registry (ChiCTR2000040804). All participants signed an informed consent form.

### Participants

Patients diagnosed with a first-ever ischemic stroke [19], confirmed by head computed tomography scanning or magnetic resonance imaging; aged≥18 years; able to understand instructions; and able to use mobile phones proficiently were included. Patients diagnosed with primary mental disorders or other serious organ dysfunction, such as liver and kidney dysfunction, were excluded.

### Interventions

Patients in the control group were provided with a health management manual with explanations and discharge advice during hospitalization; they conducted out-of-hospital follow-ups through an online WeChat public group and received monthly telephonic coaching sessions of approximately 15 minutes each, provided mainly by a qualified stroke health manager, as previously published [20]. In response to national policies, we canceled the health education courses held in our center during hospitalization to avoid social gatherings according to hospital rules.

Patients in the intervention group received 6 additional web-based educational sessions based on the HBM via Tencent Meeting, which is an audio and video conferencing application. The sessions were held every 2 weeks between 9 AM and 10 AM, with each session lasting approximately 40 minutes. The intervention was conducted in small groups, with about 8 to 10 people in each group. A qualified stroke health manager, skilled in health education, facilitated all the sessions. The first session was focused on perceived susceptibility. Approximately 20 minutes were allocated for free discussion to encourage participants to identify and express their own risk factors. The second session focused on perceived severity on the basis of the model. The third session focused on perceived benefits. Participants could ask questions and discuss the possible benefits of their behaviors. The fourth and fifth sessions mainly supplemented cues to actions. Participants were encouraged to put forward questions that they did not fully understand and received prompt explanations. The last session aimed to address patients’ perceived barriers to secondary prevention behaviors. Participants worked with others to correct incorrect beliefs and provide strategies to overcome these barriers (Table 1).

Participants received a notification on their mobile phones 1 hour before each session. We also implemented online reminders before each session and ensured that all participants attended the meetings on time. Patients who completed all sessions and participated in 3 or more discussions during the sessions were awarded an electronic version of the “Learning Star” honorary certificate to encourage them to continue to adhere to healthy behaviors.

**Table 1.** The education session structure based on the health belief model.

Intervention theme	Intervention targets	Procedure of sessions
Perceived susceptibility	Aware of the severity of the disease, current bad behaviors, and risk factors	Through the ”Screen Image Sharing“ technology of the Tencent Meeting, the PowerPoint was presented to the patients at the same time as the lecture, describing stroke-related statistics and common risk factors (eg, hypertension, diabetes, and dyslipemia).
Perceived severity	Recognize the possibility and risk of stroke recurrence	Describing the possible physical and psychological impact and economic burden of stroke recurrence through several cases that failed to engage in secondary preventive strategies and encouraging participants to share some feelings of distress, when they experienced their first stroke.
Perceived benefits	Aware of the benefits of changing daily lifestyle and following secondary prevention measures	A patient who had been followed up for 1 year without stroke recurrence and had a better prognosis was invited to share his experiences, particularly some lifestyle changes and daily disease monitoring behaviors after discharge.
Cues to actions	Master the specific ways to change the bad lifestyle	Explaining the specific methods of smoking cessation, weight control, reasonable diet, as well as daily monitoring of blood pressure and glucose, and giving medication guidance.
Cues to actions	Recognize the importance of proper use of drugs	Following the medication regimens and not stopping the medicine on their own. Highlighting the strong correlation between dyslipidemia and recurrence during the course of treatment to correct patients’ misconceptions.
Perceived barriers	Address the barriers to perform secondary prevention behaviors	Participants were asked to come up with and discuss beliefs, attitudes, or facts that may prevent them from engaging in behaviors correctly.

### Outcome Indicators

The outcome assessor was a full-time physician who was professionally engaged in the follow-up of cerebrovascular disease and was not involved in any of the program implementations. Data on patients’ sociodemographic characteristics, complications, and stroke severity as well as BP, HbA_1c_, and LDL-C results were retrieved from electronic medical records. The above physiological and biochemical indicators were collected twice: within 48 hours of admission and during the face-to-face outpatient clinic follow-up 3 months after stroke onset.

The primary outcomes were control of BP, HbA_1c_, and LDL-C levels according to the recommendations for treatment targets in the Chinese Stroke Association guidelines [21]. The reference treatment targets for patients with ischemic stroke were as follows: BP<140/90 mmHg, LDL-C<2.6 mmol/L, and HbA_1c_≤7%. BP of the nonhemiplegic side was measured after the patient rested for 5 minutes in a sitting position using a digital sphygmomanometer (Omron HBP-9020). The mean value of 3 consecutive measurements with 1-minute intervals was taken as the final result. LDL-C concentration and blood levels of HbA_1c_ were measured in fresh venous blood samples.

The outcome of self-reported medication adherence was measured by the Chinese version of the 8-item Morisky Medicine Adherence Scale (MMAS) [22,23,24]. The total score ranges from 0 to 8, with a score ≥6 classified as adherent [25]. We also used the modified Rankin Scale (mRS) to evaluate clinical outcomes in patients with stroke. mRS is a 7-level scale, ranging from 0 (no symptoms) to 6 (death), for assessing the recovery state of neurological function. Patients with an mRS score of 0-1 were defined as having favorable outcomes.

### Statistical Analyses

An a priori sample size calculation was performed for this study based on the outcome “the difference in BP at 3 months after discharge in patients with hypertensive ischemic stroke receiving a comprehensive reminder system intervention [26].” Assuming a 20% drop-out rate, at least 170 patients in each group were included to achieve a power of 0.90 with a 2-sided significance level of 5%. Baseline characteristics were reported as means (SDs), median (IQRs), and proportions, as appropriate. Means or probability and 95% CIs were used to report the differences between groups and were examined using the chi-square test for categorical variables and student 2-tailed *t* test or Mann-Whitney *U* test for continuous variables, respectively. Binary logistic regression was used to adjust for confounding variables and compare the proportion of vascular risk factors that reached the target value between the intervention and control groups. Differences in the levels of vascular risk factors between the 2 groups were compared using multiple linear regression, and clinical baseline parameters were considered as covariates. Two-sided *P* values less than .05 were regarded as statistically significant. Data analysis was performed using IBM SPSS Statistics (version 22.0; IBM Corp).

## Results

**Figure 1.**
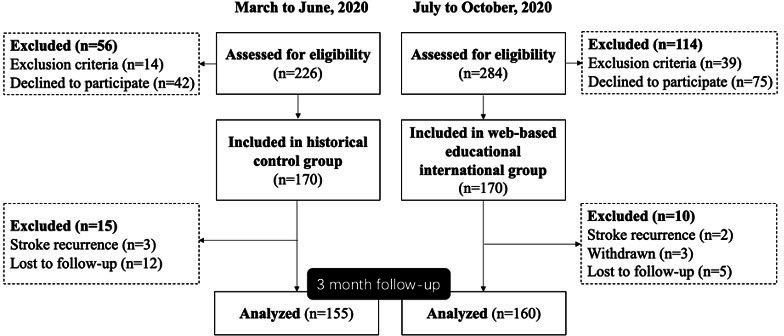
Study flow chart.

In total, 340 consecutive patients were included in this study. Figure 1 illustrates the study flow chart; 315 patients were finally analyzed, with 155 in the historical control group and 160 in the intervention group. Table 2 presents the demographic and clinical characteristics of the participants. The mean age of participants was 59.2 (SD 10.5) years (the maximum age was 88 years, and the minimum age was 25 years). There were no significant differences in participants’ age, gender, educational level, household income, and health insurance between the 2 groups. Slightly more patients in the control group had comorbidities and were smokers, but this difference was not statistically significant. There were no significant differences between the 2 groups in BP, LDL-C, and HBA_1c_ levels or in the proportion of patients who met treatment targets within 48 hours of admission (Table 3).

**Table 2.** Sociodemographic and clinical characteristics of participants (N=315).

Characteristics		Control group (n=155)	Intervention group (n=160)	*P* value
Age (years), mean (SD)		58.9 (10.3)	59.5 (10.6)	.61
**Gender, n (%)**				.50
	Male	124 (80)	123 (76.9)	
	Female	31 (20)	37 (23.1)	
**Educational level, n (%)**				.47
	High school or above	46 (29.7)	41 (25.6)	
	Middle school	41 (26.5)	52 (32.5)	
	Elementary school	68 (43.9)	67 (41.9)	
**Monthly per capita family income (RMB^a^), n (%)**				.29
	<1000	26 (16.8)	19 (11.9)	
	1000-3000	72 (46.5)	81 (50.6)	
	>3000	57 (36.8)	60 (37.5)	
**Health insurance, n (%)**				.23
	Yes	142 (91.6)	152 (95)	
	No	13 (8.4)	8 (5)	
Baseline NIHSS^b^ score, median (IQR)		2 (0-5)	2 (0-6)	.19
**Comorbidities, n (%)**				
	Hypertension	112 (72.3)	104 (65)	.17
	Diabetes mellitus	46 (29.7)	40 (25)	.35
	Hyperlipidemia^c^	96 (61.9)	85 (53.1)	.11
	Atrial fibrillation	8 (5.2)	15 (9.4)	.14
Smoking, n (%)		96 (61.9)	91 (56.9)	.36
Alcohol use^d^, n (%)		78 (50.3)	88 (55)	.41

a 1000 RMB is about US $149.

b NIHSS: The National Institutes of Health Stroke Scale.

c Hyperlipidemia is defined as elevated levels of triglyceride, total cholesterol, low-density lipoprotein cholesterol, and reduced high-density lipoprotein cholesterol levels.

d Alcohol use was defined as current or previous habitual consumption of alcohol.

**Table 3.** Level of vascular risk factors and proportions of treatment targets achieved at baseline.

Parameter	Control group (n=155)	Intervention group (n=160)	*t* test/*χ*^2^ (*df*)	*P* value
SBP^a^ (mmHg), mean (SD)	149.2 (20.5)	148.6 (23.2)	0.266 (310)^b^	.79
DBP^c^ (mmHg), mean (SD)	87.0 (11.6)	86.6 (14.4)	0.310 (303)^b^	.76
BP control, n (%)	48 (31.0)	48 (30.0)	0.035 (1)^d^	.85
LDL-C^e^ (mmol/L), mean (SD)	2.5 (0.7)	2.6 (0.8)	−1.625 (313)^b^	.11
LDL-C control, n (%)	88 (56.8)	83 (51.9)	0.761 (1)^d^	.38
HbA_1c_^f^ (%), mean (SD)	6.3 (1.4)	6.2 (1.4)	0.306 (313)^b^	.76
HbA_1c_ control, n (%)	126 (81.3)	133 (83.1)	0.181 (1)^d^	.67

a SBP: systolic blood pressure.

b Groups were compared by *t *test.

c DBP: diastolic blood pressure.

d Groups were compared by *χ*2 test.

e LDL-C: low-density lipoprotein cholesterol.

f HbA_1c_: hemoglobin A_1c_.

After intervention, participants in the intervention group were 1.93 times more likely to have controlled BP (adjusted odds ratio [aOR] 1.93, 95% CI 1.15-3.24; *P*=.012; Table 4); more patients (133/160, 83.1%) had controlled LDL-C compared to those in the control group (105/155, 67.7%; aOR 2.66, 95% CI 1.51-4.69; *P*=.001), and the proportion of patients who had an HbA_1c_ level less than 7.0% at 3 months was 91.9% (147/160) in the intervention group compared with 83.9% (130/155) in the control group (aOR 3.37, 95% CI 1.09 to 10.47; *P=*.04).

**Table 4.** Proportions achieving vascular risk factor control, medicine adherent and favorable functional outcome at 3 months.

Variables	Control group (n=155), n (%)	Intervention group (n=160), n (%)	Crude OR^a^ (95% CI)	*P* value	Adjusted OR (95% CI)	*P* value
BP^b^ control	44 (28.4)	67 (41.9)	1.82 (1.14-2.91)	.01	1.93 (1.15-3.24)^c^	.01
LDL-C^d^ control	105 (67.7)	133 (83.1)	2.35 (1.38-4.00)	.002	2.66 (1.51-4.69)^c^	.001
HbA_1c_^e^ control	130 (83.9)	147 (91.9)	2.18 (1.07-4.43)	.03	3.37 (1.09-10.47)^c^	.04
Medicine adherent^f^	98 (63.2)	127 (79.4)	2.24 (1.35-3.70)	.002	2.31 (1.37-3.87)^g^	.002
Favorable functional outcome	97 (62.6)	104 (65)	1.14 (0.72-1.81)	.57	1.22 (0.74-2.02)^h^	.44

a OR: odds ratio.

b BP: blood pressure.

c Adjusted for age, sex, cormorbidities, smoking, alcohol use, and proportion of reaching target value at baseline.

d LDL-C: low-density lipoprotein cholesterol.

e HbA_1c_: hemoglobin A_1c_.

f Medicine adherent was assessed with the Morisky Medicine Adherence Scale (MMAS). The MMAS-8 Scale (U.S. Copyright Registration No. TX0008632533), content, name, and trademarks are protected by US copyright and trademark laws. Permission for use of the scale and its coding is required. A license agreement is available from MMAR, LLC., www.moriskyscale.com.

g Adjusted for age, sex, cormorbidities, educational level, income, and insurance.

h Adjusted for age, sex, cormorbidities, smoking, alcohol use, and the National Institutes of Health Stroke Scale at baseline.

Systolic BP showed a significant reduction at 3 months (adjusted β −3.94, 95% CI −7.18 to −0.60; *P*=.02; Table 5) for the HBM-based planned web-based educational intervention program, but there was no effect on diastolic BP level (adjusted β −2.45, 95% CI −5.06 to 0.17; *P*=.07). There was also a significant postintervention reduction in the mean level of LDL-C (adjusted β −0.21, 95% CI −0.37 to −0.06; *P*=.008) and HbA_1c_ (adjusted β −0.27, 95% CI −0.37 to −0.16; *P*<.001) among the patients in the intervention group compared to those in the comparison group.

Self-reported adherence scores for the intervention and control groups at 3 months were 6.7 (SD 1.5) and 6.2 (SD 1.7), respectively (*P*=.009). The proportion of patients reporting adherence to their medication was 79.4% (127/160) in the intervention group compared to 63.2% (98/155) in the control group (aOR 2.31, 95% CI 1.37-3.87; *P*=.002). However, there was no significant difference in the favorable functional outcomes between the two groups at 90 days.

**Table 5.** Differences of vascular risk factors parameter values between the two groups at 3 months.

Parameter	Control group (n=155)	Intervention group (n=160)	Adjusted β (95% CI)^a^	*P* value
SBP^b^ (mmHg), mean (SD)	144.5 (16.7)	140.1 (16.8)	−3.94 (−7.28 to −0.60)	.02
DBP^c^ (mmHg), mean (SD)	85.3 (9.9)	82.1 (10.5)	−2.45 (−5.06 to 0.17)	.07
LDL-C^d^ (mmol/L), mean (SD)	2.2 (0.8)	2.1 (0.6)	−0.21 (−0.37 to −0.06)	.008
HbA_1c_^e^ (%), mean (SD)	6.2 (1.2)	5.9 (1.0)	−0.27 (−0.37 to −0.16)	<.001

a Adjusted for age, sex, cormorbidities, smoking, alcohol use, and baseline value.

b SBP: systolic blood pressure.

c DBP: diastolic blood pressure.

d LDL-C: low-density lipoprotein cholesterol.

e HbA_1c_: hemoglobin A_1c_.

## Discussion

### Principal Findings

Our study demonstrated that a planned web-based education program based on the HBM model positively impacted the control of vascular risk factors in patients who had ischemic stroke and improved medication adherence. The theory-guided practice may help enhance the effectiveness of health management. A comprehensive reminder system based on the HBM also improved medication adherence and reduced BP of patients who had a stroke [26]. However, in the SMS4Stroke study, guided by the HBM with behavior change theory, BP did not show a significant change between the 2 groups [27]. A possible reason is that the intervention duration was relatively short at 2 months. The HBM predicts the impact on human behavior from 6 aspects, and participants may change their behavior through health education related to these themes.

Hypertension control is a cornerstone of secondary stroke prevention. Health education based on the HBM increased the BP control compliance rate of participants by 2 times at 3 months, but the level was still suboptimal compared with other studies [26,28]. One of the reasons may be that the cold weather in the northeast and the residents’ preference for pickles led to a BP level of 149/87 mmHg at baseline, which was significantly higher than that of patients from Southern and Northern China [29,30]. However, our data are almost consistent with those of the China National Stroke Registry, a multicenter database covering the whole country [31]. Therefore, there is room for improvement in the next period.

Higher LDL-C levels have been associated with stroke recurrence over the past 10 years. Unlike hypertension and hyperglycemia, dyslipidemia may not cause significant physical discomfort for patients. In addition, a lack of knowledge about statins also makes patients likely to ignore adherence to long-term use of this medicine [32,33]. After the web-based education intervention, LDL-C levels decreased to 0.6 mmol/L (95% CI −0.7 to −0.4), which was double the 0.3 mmol/L reported in a 12-week exercise and education program for nondisabling stroke at 6 months follow-up [34]. HbA_1c_ can objectively reflect the average glycemic control over the last 3 months. With the cues to actions we provided, through lifestyle changes and adherence to hypoglycemic agents, the mean HbA_1c_ of patients with diabetes in the intervention group fell within the normal range, and 148/160 (92.5%) patients achieved control goals, which was better than the results of a 3-month poststroke education delivered via 5-minute movies to stroke survivor and caregiver dyads in Pakistan [35]. This is likely because only a quarter of the patients in this study had diabetes, and the patients had better glycemic control at baseline.

The 3-months medication adherence in these 2 groups was higher than that of previous studies [9,36]. However, it is still lower than the 89.0% medication adherence reported in a study of 600 patients conducted in 3 stroke centers across Korea [37]. Only patients who continued to take all prescribed medications were further evaluated for adherence, resulting in the exclusion of patients who reported discontinuing all or some medications, which may explain this difference. A study conducted in 2020 found that the imposed COVID-19 lockdown is unlikely to affect the medication behavior of patients with high preestablished adherence [38]. Therefore, we purposefully established the medication beliefs of patients based on the HBM model, which helps to ensure patients’ medication adherence under the normalization of the pandemic.

Although statistically significant improvements were found in both vascular risk factor control and medication adherence in the intervention group compared with the control group, the functional outcomes were not statistically different. This is probably because the neurological condition was not severe in our participants. Multicenters with large samples and multifaceted efforts are required to prove the efficacy of our web-based health education program.

Our study has several limitations. Due to the shortage of medical staff and researchers during the lockdown, this study used a historical control method, which provides less quality evidence compared to randomized controlled trials. Outcomes after intervention were evaluated for 3 months, and long-term effects need to be further assessed. The study was conducted in a single center, and the included patients may have regional characteristics, having a certain impact on the results. We only investigated overall medication adherence, and the persistence of a specific medication was unknown. The assessment methods of self-report may have led to recall bias; a more objective measure of adherence would be preferable to confirm our findings. However, this study design allowed us to realize the potential benefits of web-based health education under real-life conditions and complement evidence from randomized controlled trials.

### Conclusions

This study found that a web-based health education program under the HBM model during the COVID-19 lockdown in autumn 2020 in northeast China, designed to teach patients who experienced a stroke about secondary prevention, may result in improved vascular risk factors and medication adherence. It provides insights into achieving secondary stroke prevention goals, recommended in real-world guidelines. It also serves as a reference for the use of telemedicine to conduct disease prevention under the “new normal” of COVID-19 and the shortage of medical staff.

## Abbreviations

aOR: adjusted odds ratio
BP: blood pressure
HbA_1c_: hemoglobin A_1c_
HBM: health belief model
LDL-C: low-density lipoprotein cholesterol
MMAS: Morisky Medicine Adherence Scale
mRS: modified Rankin Scale
