# Correction: Effects of a Planned Web-Based Educational Intervention Based on the Health Belief Model for Patients With Ischemic Stroke in Promoting Secondary Prevention During the COVID-19 Lockdown in China: Quasi-Experimental Study

**DOI:** 10.2196/60953

**Published:** 2024-06-28

**Authors:** Zhuo Liu, Xin Sun, Zhen-ni Guo, Ye Sun, Yi Yang, Xiuli Yan

**Affiliations:** 1 Stroke Center, Department of Neurology, The First Hospital of Jilin University, Changchun, China; 2 Neuroscience Research Center, Department of Neurology, The First Hospital of Jilin University, Changchun, China

In “Effects of a Planned Web-Based Educational Intervention Based on the Health Belief Model for Patients With Ischemic Stroke in Promoting Secondary Prevention During the COVID-19 Lockdown in China: Quasi-Experimental Study” (JMIR Mhealth Uhealth 2024;12:e44463) the authors noted one error.

In the “Ethical Considerations” section, the following sentence:

*The study protocol was reviewed and approved by the ethical committee of the First Hospital of Jilin University (20K056-001) and was registered in the Chinese Clinical Trial Registry (ChiCTR2000040804).*

Has been changed to read as follows:

*The study protocol was reviewed and approved by the ethical committee of the First Hospital of Jilin University (20K055-001) and was registered in the Chinese Clinical Trial Registry (ChiCTR2000040804).*

The correction will appear in the online version of the paper on the JMIR Publications website on June 28, 2024, together with the publication of this correction notice. Because this was made after submission to PubMed, PubMed Central, and other full-text repositories, the corrected article has also been resubmitted to those repositories.

